# Screening high symbiotic matching combinations of rhizobia and alfalfa varieties in saline soil

**DOI:** 10.3389/fpls.2026.1953432

**Published:** 2026-10-16

**Authors:** Liying Liu, Yinghao Liu, Hongmei Liu, Hui Yan, Yiwen Yang, Xing Li, Yuzhi Wang, Zhigang Liu, Meiling Hou, Da Li

**Affiliations:** 1 Inner Mongolia Academy of Forestry Science, Hohhot, China; 2 Institute of Grassland Research of CAAS, Hohhot, China; 3 Inner Mongolia Autonomous Region Environmental Monitoring Station Ordos Sub-station, Ordos, China; 4 College of Life Science, Baicheng Development Research Institute, Baicheng Normal University, Baicheng, China; 5 Baicheng Institute of Animal Husbandry, Baicheng, China; 6 College of Grassland Science, Gansu Agricultural University, Lanzhou, China

**Keywords:** *Medicago sativa* L., nutritional quality, rhizobium, soil indicator, symbiotic matching combination

## Abstract

Rhizobium inoculation can improve alfalfa performance, but responses depend on both plant genotype and growing environment. We first screened 14 rhizobial strains plus one commercial inoculant with *Medicago varia* ‘Caoyuan No. 3’ (CY3) in a greenhouse pot experiment, together with an uninoculated control (16 treatments; three pots per treatment). Based on plant height, root length, leaf number, nodule number, and above- and belowground biomass after two months, SJN, HH, and HK were selected as higher-performing strains, whereas XBC, SWL, and XH were retained as lower-performing comparators. These six strains were then evaluated with CY3, Medicago sativa ‘Aohan’ (AH), and *M. sativa* ‘Adrenalin’ (ADN) in a replicated field experiment. Individual growth and nutritional responses varied among varieties and growth stages. At flowering, XH increased CY3 above-ground biomass by 113% relative to CK, whereas the reported TOPSIS composite ranking placed CY3+XBC first overall. The corresponding composite rankings placed AH+SJN and ADN+SWL first; notably, CK ranked second for ADN. Thus, ‘optimal’ refers to the highest reported composite score across the measured growth, nodulation and nutritional indicators, not superiority for every individual trait. Because symbiotic nitrogen fixation was not measured directly and a pre-sowing physicochemical baseline for the field soil was unavailable, the findings should be interpreted as context-dependent agronomic responses rather than direct quantification of nitrogen fixation or remediation of saline soil.

## 1 Introduction

The significant contribution of biological nitrogen fixation by agricultural systems presents a sustainable and cost-effective source of bioavailable nitrogen and is important to maintain global food security (Yu et al., 2018). Nitrogen fixation performed by symbiotic legumes and rhizobia is highly effective, with a global annual nitrogen fixation capacity estimated as 200–322 kg/hm^2^, accounting for 65% of the total biological nitrogen fixation (Mohammed et al., 2022). Rhizobia are Gram-negative bacteria abundantly present in soil that induce the formation of nodules in plant roots (Thompson et al., 2024). Rhizobia can improve the yield and quality of leguminous plants, but they also effectively improve the physical and chemical properties and fertility of the soil, thereby stimulating the growth of later crops. However, if the legume lacks a matching rhizobium strain, its utilization of nitrogen from the air will be reduced, and instead more nitrogen will be absorbed from the soil, resulting in a reduced nitrogen content of the soil and a decline of soil fertility (Schwember et al., 2019).

Alfalfa (*Medicago sativa* L.) is one of the most important feed crops in the world, with high productivity, wide adaptability, high nutritional quality and palatability; it is also an ecologically important crop, due to its ability to prevent soil erosion and improve soil fertility (Zhao et al., 2020). In China, alfalfa cultivation is mainly concentrated on the marginal soils in the northern parts of the country, where soil conditions are very poor. The challenge for farmers and researchers is to cultivate alfalfa with high yield and quality under such conditions. One key factor to influence is the symbiotic relationship between alfalfa and its rhizobium, which provides a major nitrogen source for the plants (Brambilla et al., 2024). The rhizobium not only improves the nutrient absorption capacity of alfalfa, but also increases its stress resistance, and by inhibiting competitive pathogenic microorganisms, it protects the plants from environmental damage and increases their yield (Batnini et al., 2023). An active rhizobium can improve the nutritional value of alfalfa by increasing its nitrogen, protein and nutrient content. Moreover, the co-existence of rhizobium and alfalfa can significantly improve local soil conditions (Horváth et al., 2023).

With other legumes, alfalfa is known for its specific selection of rhizobial constituents, and this preference may differ between alfalfa varieties. Thus, the beneficial symbiotic effects can vary greatly depending on a particular alfalfa variety and rhizobium combination: the amount of fixed nitrogen by alfalfa/rhizobium symbiosis ranges from 0 to 210 kg N/hm^2^/year, depending on the bacterial strain and plant variety (Checcucci et al., 2018). Compared with a poorly matching alfalfa/rhizobium combination, a better match can promote plant growth, root development and nitrogen accumulation, as well as improve soil N availability and soil fertility (Schumacher et al., 2025). Specific interactions between the plant and its rhizobium determine the efficiency of the match. This coupled with the presence of a large number of competitive but less effective indigenous rhizobium bacteria in the soil, adds to the challenge of improving symbiotic nitrogen fixation for increasing yields. This challenge can be overcome by introducing suitable commercial inoculants into the soil. The matching effect and interaction mechanism between alfalfa varieties and their rhizobia need to be explored in depth, whereby major local cultivars need to be supplied with the optimal rhizobial strains that are correctly matched. This is of great importance for developing sustainable forage production practices and ensuring food security (Cobos-Porras et al., 2021).

In this study, 14 field-isolated rhizobial strains and one commercial inoculant were first screened with CY3 under greenhouse conditions. Six strains representing contrasting greenhouse performance were then tested with three alfalfa varieties in the field. We evaluated plant growth, nodulation, forage nutritional indicators, and selected soil properties to identify promising variety–strain combinations and to determine whether greenhouse performance was retained under field conditions.

## 2 Materials and methods

### 2.1 Plant and rhizobium materials

Three alfalfa varieties were included. According to the germplasm supplier records, ‘Caoyuan No. 3’ (CY3) was treated as Medicago varia Martin., whereas ‘Aohan’ (AH) and ‘Adrenalin’ (ADN) were treated as *Medicago sativa* L. Collective references to the three entries therefore use the common term ‘alfalfa’ rather than assigning all three to *M. sativa*. The seed materials were provided by Inner Mongolia Agricultural University.

Fourteen rhizobial from Key Laboratory of Forage Cultivation, Processing and High Efficient Utilization of Ministry of Agriculture and Rural Affairs, Inner Mongolia Agriculture University, and their plant hosts are marked in the table (**Table 1**). A commercial rhizobial mixture (DZ; Beijing Crowo Ecological Technology Co., Ltd.) was also included.

**Table 1:** Host plants from which rhizobium strains were isolated.

Plant host	*Sinorhizobium kummerowiae* strain CCBAU71714	*Rhizobium giardinii* strain H152	*Sinorhizobium meliloti* strain IAM12611	*Sinorhizobium saheli* strain NBRC100386	*Sinorhizobium meliloti* strain NBRC14782	*Rhizobium giardinii* strain H152
*Medicago sativa* L. ‘ MagnumVII’	SJN					
*Medicago falcata* (L.) cv. Hulunbeier	H2					
*Medicago officinalis* (L.) Desr	HH				XH	
*Medicago albus* Desr.	XBC		XK	HK		
*Medicago sativa* (L.) cv. Kangsai			DK			
*Medicago sativa* varia Martin cv. Caoyuan No.3		DC3	H7			
*Medicago sativa* (L.) cv. Deguo				SD		
*Medicago sativa* (L.) cv. WL319					SWL	
*Medicago sativa* (L.) cv. Xinjiangdaye						H8

### 2.2 Pot experiments

Field soil for the greenhouse substrate was collected from the experimental site of Inner Mongolia Agricultural University, Hohhot, China (111°22′30″ E, 40°41′30″ N). The site had not previously been cropped or fertilized. Soil was air-dried, passed through a 2-mm sieve, and homogenized. It had a pH of 8.6, electrical conductivity of 4.2 dS cm^−1^, and organic matter content of 12.02 g kg^−1^. Total N, total P, available N, available P, available K, and total K were 1, 370 mg kg^−1^, 1, 100 mg kg^−1^, 106.4 mg kg^−1^, 53.25 mg kg^−1^, 46.79 mg kg^−1^, and 9.18 g kg^−1^, respectively. Because the measured EC is well below the conventional threshold used to define saline soil, this substrate is described here as alkaline rather than saline. Vermiculite, volcanic rock, and soil were autoclaved twice for 30 min at 120 °C. Each 10 × 10 cm pot contained 150 g volcanic rock, 75 g vermiculite, and 25 g sterilized soil. Moisture was adjusted to 70% of saturation and allowed to equilibrate overnight.

CY3 was used in the greenhouse screening. Seeds were scarified with sandpaper, rinsed with sterile water, surface-sterilized in 5% NaClO for 8 min, and washed thoroughly with sterile water. Seeds were kept in darkness at 4 °C for 2 days, incubated in darkness at 25 °C for another 2 days, and then maintained at room temperature for 1 day. The 14 field isolates and the commercial inoculant were activated individually on YMA agar and transferred to liquid YMA medium at 28 °C and 220 rpm for 2–4 days until OD_600_ reached 0.8–1.0.

Forty-eight pots were assigned to 16 treatments (14 field isolates, one commercial inoculant, and one uninoculated control), with three replicate pots per treatment. Uniform seedlings were immersed in the corresponding bacterial suspension; sterile water was used for the control. Five seedlings were transplanted into each pot, and 1 mL of bacterial suspension was applied around each seedling (5 mL pot^−1^). Greenhouse temperatures were 24–33 °C during the day and 16–22 °C at night, with 60–85% relative humidity. Pots were watered with sterile water (pH 7.1) every 2 days to maintain approximately 70% soil moisture.

After two months, plant height, root length, leaf number, and nodule number per plant were measured following Andrzejewska et al. (2020) and Abid et al. (2016). Above- and belowground tissues were dried at 80 °C for 16 h and weighed. The six field-test strains were selected to represent contrasting greenhouse responses: SJN, HH, and HK were the higher-performing group, whereas XBC, SWL, and XH were retained as lower-performing comparators.

### 2.3 Field experiments

The field experiment began in May 2025 on the campus of Inner Mongolia Agricultural University, and the relevant information for the test site is consistent with that for 2.2 pot experiment. The three higher-performing greenhouse strains and the three lower-performing comparators were evaluated with CY3, AH, and ADN, producing 18 inoculated variety–strain combinations plus one uninoculated control for each variety. Pellet-coated seeds were prepared as follows. Rhizobia were cultured in liquid YEMA medium for 4 days to OD_600_ ≈ 0.5. A peat carrier containing 488.5 g peat, 1 g sucrose, 0.5 g superphosphate, 10 g lime, 1 mL of 0.5% sodium molybdate, and 1 mL of 0.5% boric acid was autoclaved at 121 °C for 30 min. Bacterial suspension and peat carrier were mixed at 1:2. Then, 5 mL of 4% arabinose was mixed with 15 g inoculant and applied to 100 g seed until each seed was coated (reported bacterial content ≥10^9 seed−1).

The field experiment was conducted in a completely randomized block design. Sown on April 20, 2025, a continuous field area of approximately 700 m^2^ was divided into three 23 m × 10 m main plots, one for each alfalfa variety (**Figure 1**). Each main plot contained three 23 m × 3 m replicate blocks. Each block was divided into seven 3 m × 3 m treatment plots separated by 20 cm, comprising one uninoculated control and six inoculated treatments. There were 10 lines of forage in each plot with a row width of 30 cm. Seeds were drill-sown at 1–2.5 cm depth. The control was sown before the inoculated plots to reduce cross-contamination. The first harvest will begin on June 27, 2025, each treatment had three field replicates. The trial covered the 2025 growing season and was managed according to local agronomic practice.

**Figure 1:**
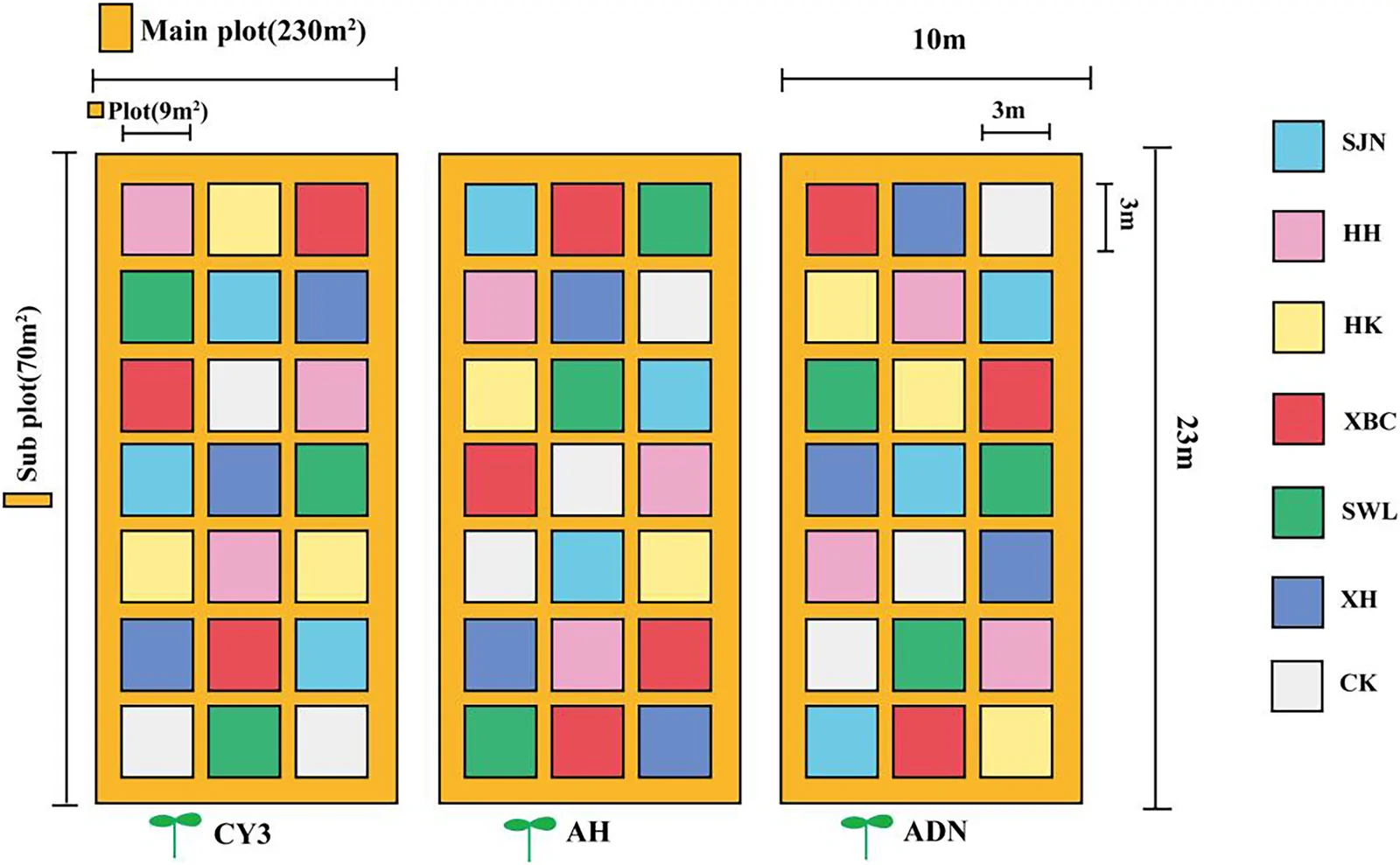
Schematic diagram of the field experimental design.

At the branching, budding, and flowering stages, plant height, branch number, aboveground biomass, nodule number, and nodule weight were measured following Andrzejewska et al. (2020) and Abid et al. (2016). Above- and belowground tissues were dried at 80 °C for 16 h before weighing. Ten plants were measured per treatment. At budding, leaf total N was determined by the Kjeldahl method, and crude protein (CP) was calculated as total N × 6.25 (Na et al., 2022). Phosphorus and potassium were measured with an M410 flame photometer (Sherwood, Cambridge, UK) (Sun et al., 2022). At flowering, rhizosphere soil was collected at 10–20 cm depth from the three combinations selected by the reported TOPSIS ranking. Soil was also collected at the same depth from unplanted plots at the end of the trial (KB). KB is an end-of-trial unplanted reference, not a pre-sowing baseline. Twelve soil samples were analyzed (four treatments × three replicates). Air-dried samples were ground, passed through a 2-mm sieve, and analyzed for pH, total carbon (TC), total nitrogen (TN), C/N ratio, total phosphorus (TP), total potassium (TK), total sulfur (TS), organic carbon (OC), available phosphorus (AP), available potassium (AK), ammonium N (AN), and nitrate N (NN) following Carneiro et al. (2021).

### 2.4 Data analysis

Statistical analyses were conducted in SPSS 26.0.0.2 (SPSS Inc., Chicago, IL, USA). For the greenhouse screen, treatments were compared by one-way ANOVA followed by Duncan’s multiple-range test. For the field data, separate one-way ANOVAs were conducted within each alfalfa variety and growth stage, followed by Duncan’s test (P< 0.05). These analyses compare strains within a defined variety and stage; they do not constitute a factorial test of variety × strain × stage interactions. With three field replicates, the inferential results should be regarded as exploratory. Graphs were prepared in Prism 8.4.3 (GraphPad Software, LLC).

In order to achieve the multiple goals of maintaining yield, improving efficiency and optimizing water consumption characteristics, the TOPSIS method was applied to assess corn under different irrigation treatments, focusing on GY, NPFP, WUE and E. The reported TOPSIS analysis ranked the seven treatments within each variety using the measured growth, nodulation, and nutritional indicators shown in **Figures 2**–**4** (Seyedmohammadi et al., 2018).

**Figure 2:**
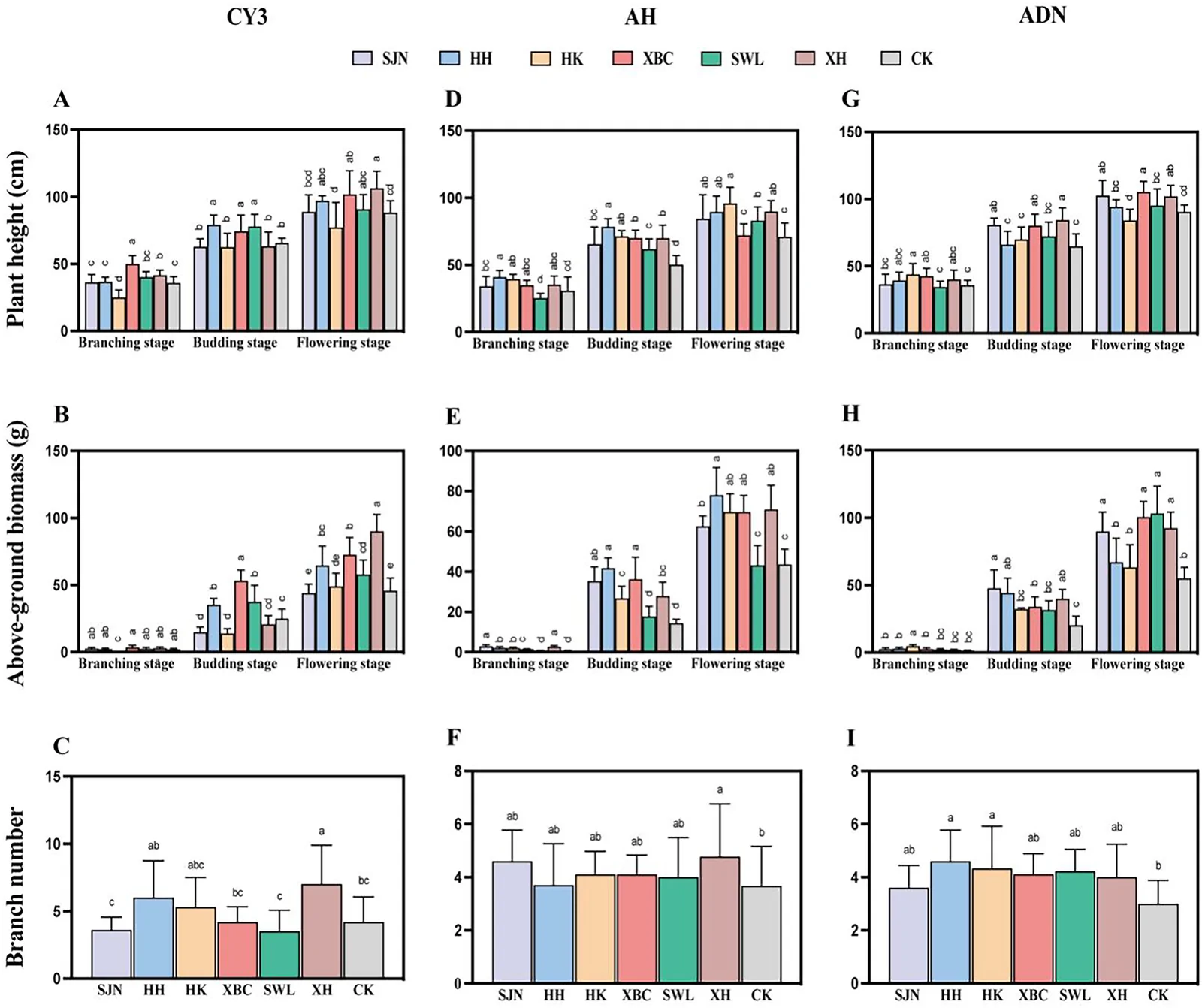
Effect of inoculation with different rhizobia on aboveground growth of three alfalfa varieties under field conditions. **(A)** Plant height of CY3, **(B)** aboveground biomass of CY3, **(C)** branch number of CY3, **(D)** plant height of AH, **(E)** aboveground biomass of AH, **(F)** branch number of AH, **(G)** plant height of ADN, **(H)** aboveground biomass of ADN, and **(I)** branch number of ADN. Separate one-way ANOVAs compared strains within each variety and growth stage. Error bars represent SD (n = 3). Different letters indicate significant differences at P< 0.05.

**Figure 3:**
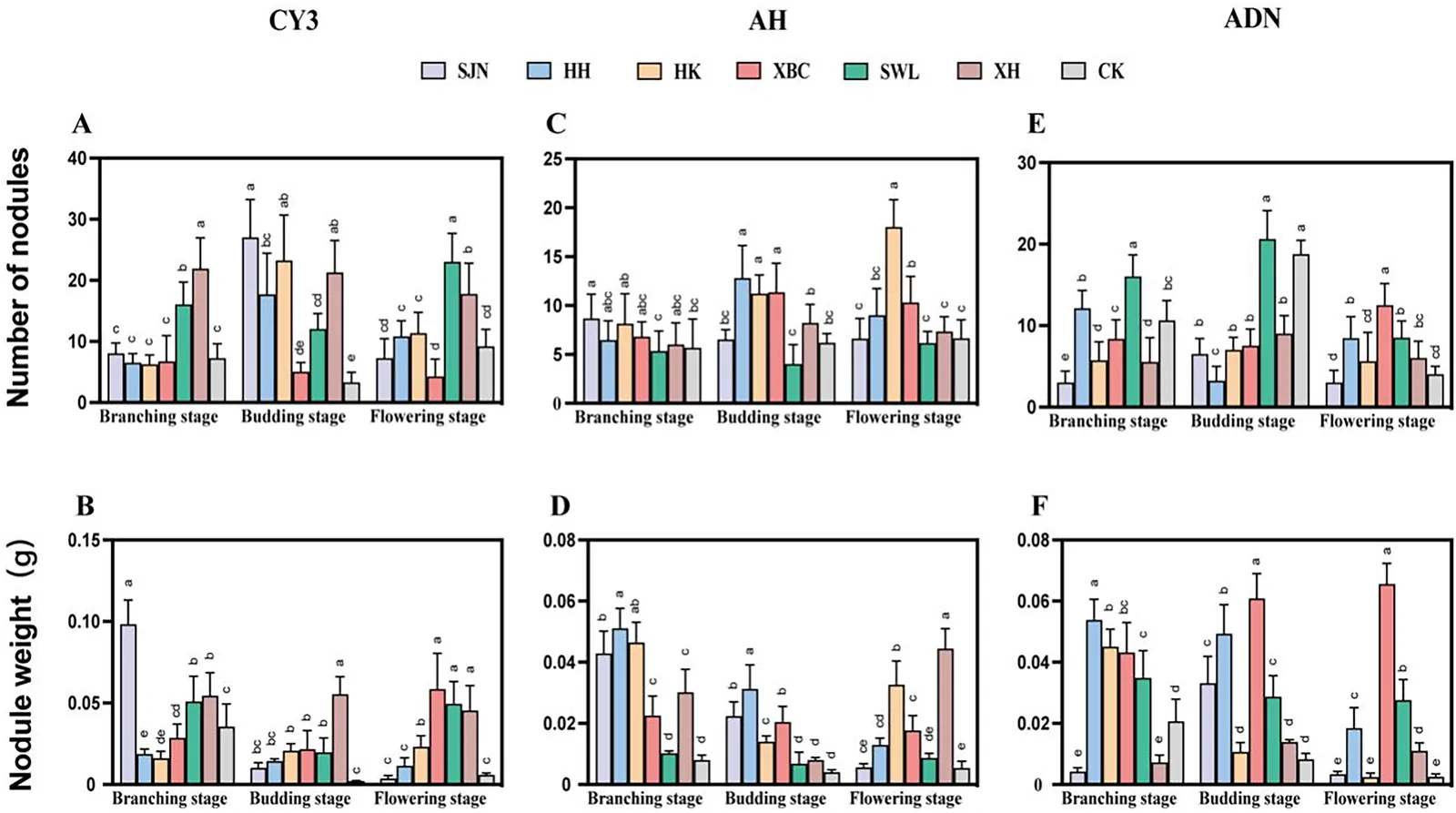
Effect of inoculation with different rhizobia on the growth of different varieties of alfalfa’s belowground parts under field experimental conditions. **(A)** The nodule number of CY3, **(B)** The nodule weight of CY3, **(C)** The nodule number of AH, **(D)** The nodule weight of AH, **(E)** The nodule number of ADN, **(F)** The nodule weight of ADN. One-way ANOVA was used to analyze the effects of different rhizobia on growth of different alfalfa varieties. Error bars represent SD values of three replicates. Different letters above the bars indicate statistically significant differences (P<0.05).

**Figure 4:**
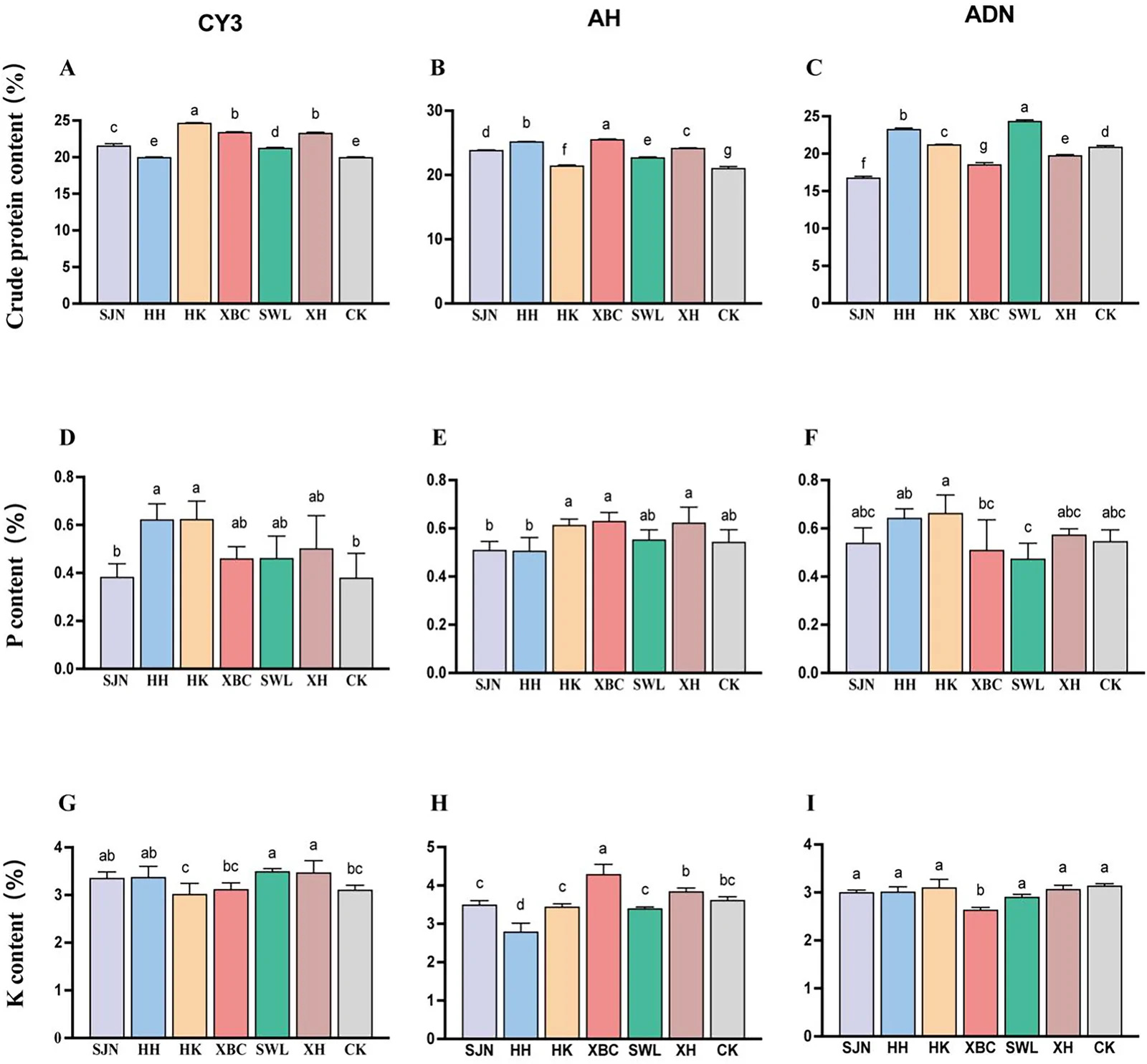
Effect of rhizobial inoculation on the nutritional quality of three alfalfa varieties under field conditions. **(A–C)** CP content of CY3, AH, and ADN, respectively; **(D–F)** P content of CY3, AH, and ADN, respectively; and **(G–I)** K content of CY3, AH, and ADN, respectively. Separate one-way ANOVAs compared strains within each variety. Error bars represent SD (n = 3). Different letters indicate significant differences at P< 0.05.

(a) Construction of the evaluation indices’ contribution matrix $P_{ij}={\left(P_{ij}\right)}_{nm}$where *P*ij denoted the jth evaluation index of the ith treatment derived from the original data, n represented the total number of treatments and m signified the number of evaluation indices m signified the number of evaluation indices.

(b) Normalize the contribution matrix.

(1) $$V_{i}^{+}=\left(P_{ij}-\min\left(P_{ij}\right)\right)/\left(\max\left(P_{ij}\right)-\min\left(P_{ij}\right)\right)$$

(2) $$V_{ij}=\left(\max\left(P_{ij}\right)-P_{ij}\right)/\left(\max\left(P_{ij}\right)-\min\left(P_{ij}\right)\right)$$

For each evaluation index, the highest positive value (*V*j+) and the lowest negative value (*V*j −) were identified accordingly.

(c) Calculating the Euclidean distance:

(3) $$S_{i}^{+}=\sqrt{\overset{m}{\underset{j=1}{\sum }}{\left(v_{ij}-v{\text{j}}^{+}\right)}^{2}}$$

(4) $$S_{i}^{-}=\sqrt{\overset{m}{\underset{j=1}{\sum }}{\left(v_{ij}-v{\text{j}}^{-}\right)}^{2}}$$

(5) $$C_{i}=\frac{S_{i}^{-}}{S_{i}^{+}+S_{i}^{-}}$$

where *S*^+^ and *S*^−^ represented the distances from the target value to the positive and negative ideal solutions, respectively.where Ci represented the index for comprehensive evaluation of benefits.

‘Optimal’ denotes the highest reported composite score and does not imply superiority for every individual indicator. The indicator-weight vector and intermediate normalized decision matrix were not available in the archived analysis records; therefore, the reported TOPSIS rankings are retained as descriptive results pending verification against the original analysis files.

## 3 Results

### 3.1 Alfalfa growth following different rhizobia inocula under greenhouse conditions

CY3 responded differently to the rhizobial inocula in the greenhouse screen. The experiment included an uninoculated control (CK) and a commercial rhizobial mixture (DZ). Plant height was greatest with SJN, followed by HH and HK, and root length was greatest with SJN, followed by HK (**Figure 5A**). H8 and SD produced the shortest plants. SJN ranked highly for both plant height and root length, whereas SWL ranked low for both traits.

**Figure 5:**
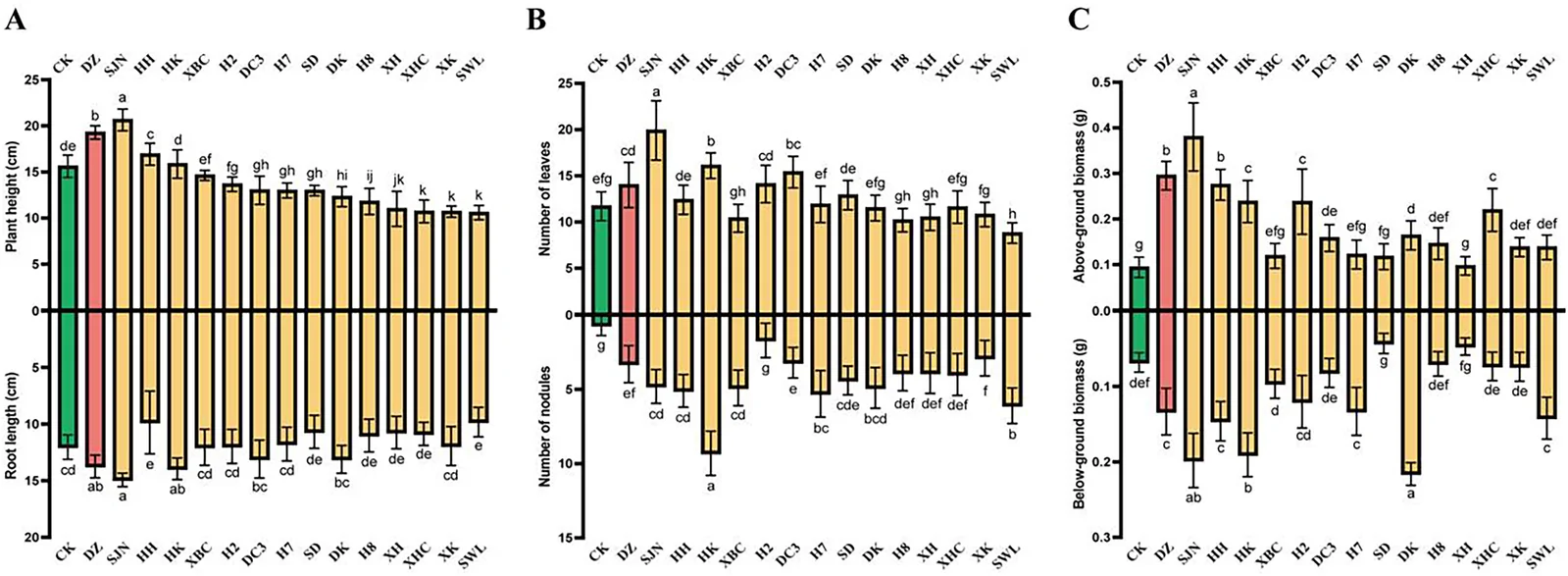
Effect of inoculation with different rhizobia on the growth of alfalfa under pot experiment conditions. **(A)** Effect on plant height and root length, **(B)** Effect on leaf count and nodule number, **(C)** Effect on above-ground and below-ground biomass. One-way ANOVA was used to analyze the effects of different rhizobia on alfalfa growth. Error bars represent SD values of three replicates. Different letters above the bars indicate statistically significant differences (P<0.05).

Leaf number exceeded CK in most inoculated treatments and was greatest with SJN, followed by HK and DC3 (**Figure 5B**). Nodule number varied among inocula but exceeded CK in all inoculated treatments; HK produced the greatest number of nodules.

Aboveground biomass was greatest with SJN and lowest with SD and XH. Belowground biomass was greatest with DK, followed by SJN and HK. SJN, HK, HH, and DK produced relatively high total biomass, whereas SD and XH did not outperform CK. Several isolates also produced less total biomass than the commercial DZ inoculant (**Figure 5C**).

On the basis of the combined greenhouse growth measurements, SJN, HH, and HK were selected as the higher-performing group for field evaluation. XBC, SWL, and XH, which showed lower greenhouse performance, were included as contrasting comparators.

### 3.2 Field experiments: rhizobium inoculation affects aboveground growth of alfalfa

The six selected strains were evaluated with CY3, AH, and ADN in the field. Their rankings differed from those in the greenhouse. For CY3, XBC produced relatively high plant height and aboveground biomass at branching and budding, whereas XBC and XH both performed well at flowering (**Figures 2A, B**). The largest single biomass response reported for CY3 was observed with XH at flowering: 53.10 g versus 24.92 g in CK, an increase of 113% (**Figure 2B**). This single-stage response should not be confused with the reported TOPSIS composite ranking, which placed CY3+XBC first overall. HK, despite being selected as a higher-performing greenhouse strain, produced the lowest CY3 plant height and aboveground biomass across the assessed field stages. CY3 branch number was greatest with XH (7.0 versus 4.2 in CK; +67%; **Figure 2C**).

For AH, plant height was greatest with HH at branching and budding and with HK at flowering (**Figure 2D**). Aboveground biomass was greatest with HH; at budding, HH produced 41.62 g compared with 14.31 g in CK (+191%; **Figure 2E**). Branch number was greatest with XH (4.78 versus 3.67 in CK; +30%), although it did not differ significantly from all other inoculated treatments (**Figure 2F**).

For ADN, SJN, XBC, and XH produced relatively high plant height at budding and flowering (**Figure 2G**). Aboveground biomass was high with SJN at budding and with SWL, XBC, XH, and SJN at flowering (**Figure 2H**). At budding, SJN produced 47.51 g compared with 20.21 g in CK (+135%). Branch number was greatest with HH (4.6) and HK (4.3), representing increases of 53% and 43%, respectively, relative to CK (3.0); however, these values did not differ significantly from all other treatments (**Figure 2I**).

Overall, the best-performing strain depended on the variety, growth stage, and response variable. XH and XBC produced strong CY3 responses; HH and HK produced strong AH responses for several growth traits; and SJN, XBC, SWL, and XH each performed well for at least one ADN growth trait. The reversal in performance of strains such as XBC between the sterilized greenhouse substrate and the nonsterile field environment demonstrates context dependence, but the present analyses do not identify a causal mechanism or formally test a strain × variety × environment interaction.

### 3.3 Effects on underground parts of alfalfa grown in the field

Nodule number and nodule weight varied with strain, variety, and growth stage (**Figure 3**). For CY3, XH produced the most nodules at branching; SJN, HK, and XH produced high nodule numbers at budding; and SWL and XH produced high values at flowering (**Figure 3A**). Nodule weight was greatest with SJN at branching, XH at budding, and XBC at flowering (**Figure 3B**). At budding, CY3+XH produced 21.25 nodules and 55.2 mg nodule weight, 5.54- and 28.05-fold the CK values, respectively. XBC did not significantly increase CY3 nodule number but increased nodule weight at flowering. For AH, HH produced 12.8 nodules and 31.2 mg nodule weight at budding, whereas HK performed well at flowering (**Figures 3C, D**). For ADN, XBC produced the greatest nodule weight at flowering, and SWL produced high nodule number and weight at budding (**Figures 3E, F**).

### 3.4 Nutritional parameters of the inoculated alfalfa grown in the field

Nutritional indicators were measured at budding (**Figure 4**). CP content was greatest with HK for CY3 (**Figure 4A**), XBC for AH (**Figure 4B**), and SWL for ADN (**Figure 4C**). P content was increased most clearly by HH and HK for CY3 (**Figure 4D**), by XBC, XH, and HK for AH (**Figure 4E**), and by HK and HH for ADN (**Figure 4F**).

K content was relatively high with SWL, XH, HH, and SJN for CY3 (**Figure 4G**) and with XBC for AH (**Figure 4H**). None of the inoculated treatments improved K content in ADN relative to CK, and XBC significantly reduced it (**Figure 4I**).

### 3.5 Screening of optimized symbiotic matching combinations of alfalfa varieties and rhizobia

The reported TOPSIS scores and ranks are shown in **Table 2**. For CY3, the composite scores for XBC, HK, HH, SWL, SJN, XH, and CK were 0.840, 0.678, 0.634, 0.760, 0.739, 0.810, and 0.621, respectively; XBC ranked first. For AH, the corresponding scores were 0.377, 0.370, 0.603, 0.342, 0.680, 0.457, and 0.492; SJN ranked first. For ADN, the scores were 0.661, 0.307, 0.327, 0.802, 0.384, 0.145, and 0.712; SWL ranked first and CK ranked second.

**Table 2:** Reported TOPSIS composite scores and ranks for alfalfa variety–rhizobium combinations under field conditions.

Treatment	CY3				AH				ADN			
	*D^+^*	*D^-^*	*C_i_*	Rank	*D^+^*	*D^-^*	*C_i_*	Rank	*D^+^*	*D^-^*	*C_i_*	Rank
XBC	0.471	0.480	0.840	1	0.115	0.303	0.377	5	0.304	0.365	0.661	3
HK	0.685	0.602	0.678	5	0.108	0.310	0.370	6	0.493	0.162	0.307	6
HH	0.388	0.569	0.634	6	0.341	0.077	0.603	2	0.452	0.182	0.327	5
SWL	0.549	0.605	0.760	3	0.080	0.338	0.342	7	0.414	0.465	0.802	1
SJN	0.524	0.654	0.739	4	0.418	0.125	0.680	1	0.321	0.239	0.384	4
XH	0.427	0.460	0.810	2	0.195	0.223	0.457	4	0.240	0.136	0.145	7
CK	0.452	0.491	0.621	7	0.230	0.188	0.492	3	0.328	0.441	0.712	2

‘Optimal’ denotes the highest reported composite score within a variety and not superiority for every individual indicator.

Accordingly, CY3+XBC, AH+SJN, and ADN+SWL were the highest-ranked combinations in the reported composite analysis. Here, ‘highest-ranked’ means the largest reported TOPSIS score across the included growth, nodulation, and nutritional indicators; it does not mean that the combination was best for every trait or growth stage. The second-place rank of CK for ADN also shows that five inoculated ADN treatments did not outperform the uninoculated control on the reported composite score. Therefore, the results do not support a general claim that every rhizobial inoculation improves ADN performance.

### 3.6 Effect of optimized symbiotic matching combination on major soil nutrient indexes

To describe soil conditions associated with the three highest-ranked combinations, rhizosphere soil was collected from CY3+XBC, AH+SJN, and ADN+SWL plots at flowering and compared with the end-of-trial unplanted reference (KB). Because no pre-sowing field-soil baseline was available, these comparisons describe treatment-associated differences and cannot establish before-to-after changes.

Soil pH was lower in the three planted/inoculated treatments than in KB (**Figure 6A**). TC, TN, TP, TK, and TS were greatest in ADN+SWL and exceeded KB in the reported comparisons (**Figures 6B, C, E–G**). The C/N ratio was lower in all three combinations than in KB (**Figure 6D**). OC was lower in CY3+XBC and ADN+SWL than in KB (**Figure 6H**). AP was higher in all three combinations than in KB (**Figure 6I**), AK was highest in ADN+SWL (**Figure 6J**), AN was highest in AH+SJN (**Figure 6K**), and NN was lower in all three combinations than in KB (**Figure 6L**). These are end-of-trial associations and should not be interpreted as direct evidence of causal soil improvement.

**Figure 6:**
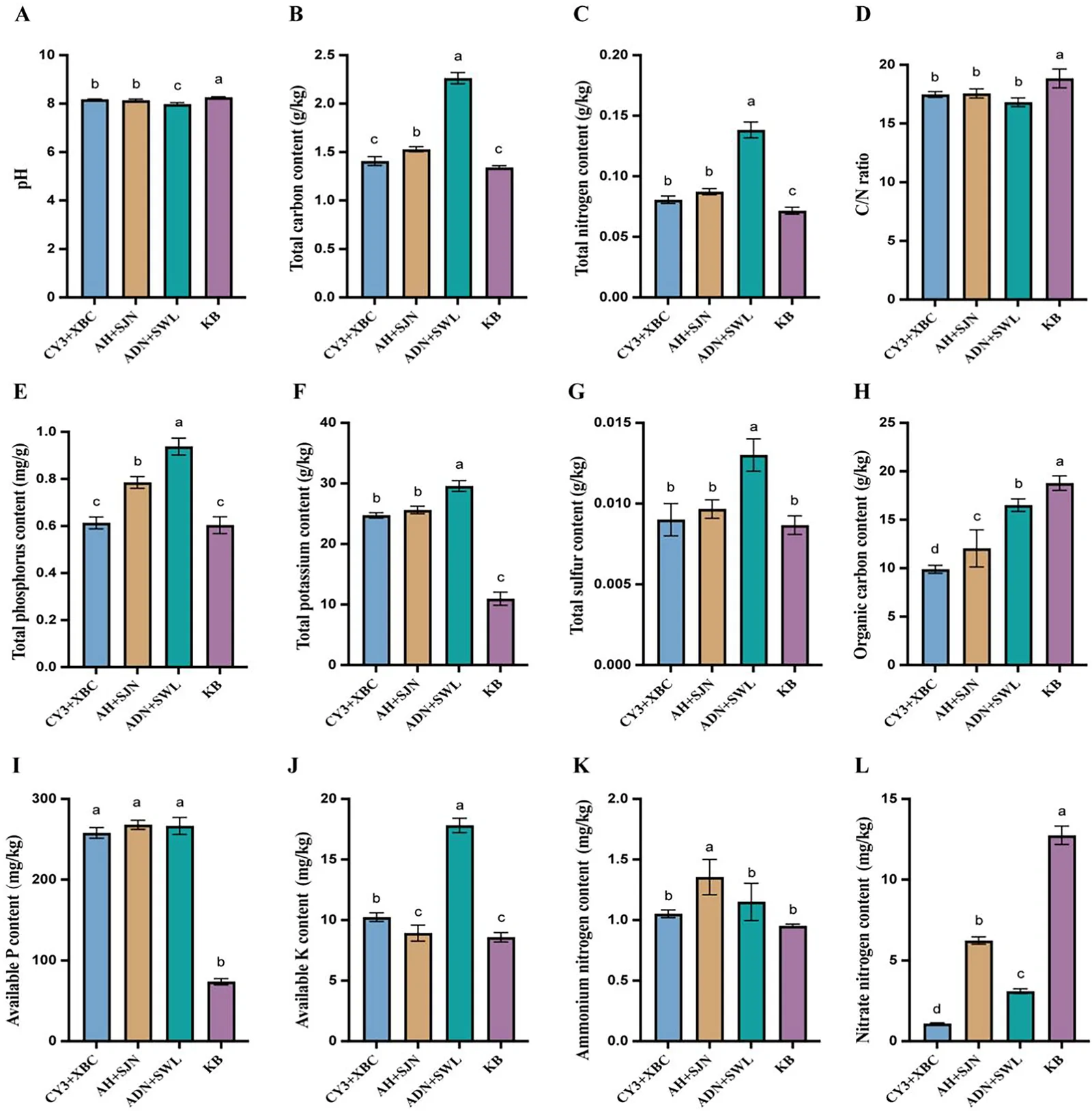
Effect of field treatments comprising each alfalfa variety and its highest-ranked rhizobial strain on end-of-trial soil physicochemical properties. **(A)** pH, **(B)** total carbon, **(C)** total nitrogen, **(D)** C/N ratio, **(E)** total phosphorus, **(F)** total potassium, **(G)** total sulfur, **(H)** organic carbon, **(I)** available phosphorus, **(J)** available potassium, **(K)** ammonium nitrogen, and **(L)** nitrate nitrogen. KB is the unplanted end-of-trial reference. Separate one-way ANOVAs compared the four treatments. Error bars represent SD (n = 3). Different letters indicate significant differences at P< 0.05.

## 4 Discussion

### 4.1 Effects of inoculation with rhizobium on growth and nutritional quality of alfalfa

The greenhouse-to-field reversal in strain performance is a central result. SJN, HH, and HK performed well in the sterilized greenhouse substrate, whereas XBC and SWL were among the lower-performing strains. In the field, XBC produced strong responses with CY3, while HK performed poorly for CY3 growth. Several nonexclusive factors could contribute: the greenhouse substrate contained sterilized vermiculite, volcanic rock, and a small quantity of sterilized alkaline soil, whereas the field soil was nonsterile and exposed to indigenous rhizobia, other microorganisms, variable nutrient availability, temperature, and water conditions. Differences in strain establishment and competition may therefore have altered host responses. Miao et al. (2018) inoculated 5 alfalfa varieties with rhizobium in the coastal areas of the Yellow River Delta, and the results showed that the technology could increase the hay yield of alfalfa by 50.3-93.2% and the amount of nodules by 70.97%-73.39%. Tang et al. (2023) inoculated alfalfa with rhizobium in 18 experimental sites in Liaoning Province, Heilongjiang Province and Inner Mongolia Autonomous Region, and the results showed that the hay yield increased by 37.8% on average, and the area with the highest increase reached 100% . Wu et al. (2022) showed that after inoculating alfalfa with rhizobium in the high cold and damp area of Gansu Province, the rate of seedling nodulation increased by 51.1%, the amount of nodulation at the branching stage increased by 9.3%, and the grass yield increased by 27%. These explanations are hypotheses, because strain colonization, microbial communities, and a pre-sowing field-soil baseline were not measured. We consequently describe the reversal as context-dependent performance rather than a demonstrated strain × variety × soil interaction.

Nodule number and weight provide evidence of nodulation but do not quantify symbiotic nitrogen fixation (Lu et al., 2017). Only a certain rhizobium combined with a certain alfalfa variety could produce higher nitrogen fixation. Inoculation with the correct match of rhizobium promotes the growth of plant root nodules that generate organic acids, reducing the fiber content of alfalfa hay and increasing its nutrient content in terms of crude protein and phosphorus content (Neu et al., 2017), but a good match between rhizobium and host is required to achieve the best nitrogen fixation efficiency for alfalfa (Lu et al., 2025). Likewise, biomass and CP are indirect response variables that can be influenced by nutrient supply and environmental conditions. Because acetylene reduction, 15N-based measurements, ureide analysis, and other direct nitrogen-fixation assays were not performed, the present study cannot compare nitrogen-fixation rates among treatments. The reported combinations should therefore be regarded as candidates for agronomic validation rather than confirmed high-efficiency nitrogen-fixing symbioses.

### 4.2 Effects of inoculating rhizobia on soil nutrients

The three highest-ranked variety–strain treatments differed from the unplanted end-of-trial reference in pH and several nutrient indicators. Soil nitrogen, phosphorus and potassium are essential elements for plant growth, and the content and proportion of the three elements are one of the most important indicators to measure soil fertility (Staudinger et al., 2016). Soil nitrogen, phosphorus and potassium content affects crop yield and quality to a large extent. Related studies show that the symbiotic nitrogen fixation between rhizobia and legumes is very sensitive to the presence of phosphorus and its content in the soil has a certain impact on the growth and development of alfalfa and nodulation, as a lack of phosphorus will inhibit the fixation and absorption of nitrogen by rhizobia (Sheng et al., 2020). Thus, the content of nitrogen, phosphorus and potassium in the soil is an important factor to take into consideration. Plant rhizosphere nitrogen content and phosphorus content can promote each other, and the increase of nitrogen can indirectly affect the availability of phosphorus, while phosphorus can also affect the retention and loss of nitrogen in soil by stimulating microbial and plant root activities (Dicenzo et al., 2015). These comparisons do not establish that inoculation caused a net improvement from the initial field condition because no pre-sowing baseline was available and the reference plots were unplanted. Potassium is another important element for plant growth (Barlas et al., 2025). In this experiment, total potassium content and available potassium content in soil were significantly increased after inoculation with rhizobia, indicating that inoculating specific rhizobia could increase total potassium content in soil and accelerate the absorption of available potassium by plants. Adomako et al. (2021) found that the application of nitrogen-fixing rhizobium and rhizosphere growth-promoting bacteria fertilizer could significantly increase the contents of total nitrogen, total phosphorus, total potassium, available phosphorus and available potassium in alfalfa soil, and significantly increase the total nitrogen content of alfalfa plants. Wang et al. (2021a) found that the contents of total nitrogen and available nitrogen in alfalfa soil increased significantly after inoculation with nitrogen-fixing rhizobium, while the contents of available phosphorus, available potassium and sulfur elements decreased significantly. There are some differences between the results of this study and those of others, which may be caused by different test materials, nitrogen-fixing strains, and soil types.Inoculation of rhizobium with high nitrogenase activity in leguminous forage can improve nitrogen fixation efficiency, promote plant growth and development, improve nutrient quality, and enhance soil fertility through biological nitrogen fixation (Hack et al., 2019). Wang et al. found that inoculation of rhizobium with red clover can significantly increase the content of organic matter and available NPK in rocky desertification soil, promote root growth of red clover, and increase the number and quality of root nodules (Wang et al., 2018). Kane found that the contents of total nitrogen, total phosphorus, total carbon and nitrate nitrogen in soil decreased and the ratio of carbon and nitrogen increased after inoculation with rhizobium. This indicated that rhizobia could promote plant growth by improving the proportion of elements in soil (Keller and Lau, 2018) At present, the direction of development of agriculture is to maximize yields. The growth promotion effect of rhizobia on plants is mainly reflected by two aspects: one is the enhancement of plants to absorb mineral nutrients in soil; the other is to improve the tolerance of plants to adversity (Lagunas et al., 2023). As demonstrated here, rhizobium inoculation can promote the absorption and utilization of nitrogen by leguminous plants such as alfalfa. Others demonstrated a higher survival rate of alfalfa plants following inoculation with rhizobium compared to a control without such treatment. These authors also observed that after inoculating alfalfa with rhizobia, the physical and chemical properties and structure of saline-alkali soil were improved, pores were enlarged, the content of water-stable aggregates was increased, and permeability was improved, which was more conducive to crop growth (Wekesa et al., 2022). Plant nutrient uptake, root inputs, rhizosphere acidification, and microbial transformations could all contribute to the observed patterns, but none was measured separately.

The observed differences in TK and AK also varied among treatments. Such variation may reflect differences in plant uptake, mineral weathering, rhizosphere chemistry, or spatial heterogeneity. The present design cannot distinguish these alternatives. We therefore report the measured differences without attributing them to a specific rhizobial mechanism.

Previous studies have proposed that rhizobial inoculation can alter rhizosphere pH, nutrient availability, and microbial communities. Wang et al. (2021b) reported that inoculation of rhizobia into the soil can improve soil properties, including a change in soil pH, water content and nitrate nitrogen content, and increase the diversity and change the composition of the soil bacterial community. Inoculation can also inhibit the proliferation of pathogenic bacteria to a certain extent, improve the plant's stress resistance, and promote additional cooperation between the plant and beneficial microorganisms (Yu et al., 2023). Further, as the inoculated bacteria enhance the growth of beneficial microorganisms that produce a variety of acidic substances that react with soil substrates, consolidated nutrients are reactivated, further increasing soil fertility (Zhang et al., 2018). However, microbial community composition, pathogen suppression, acidic metabolite production, and strain persistence were not measured here. These mechanisms remain testable hypotheses for future work rather than conclusions of the present experiment.

Taken together, the data support variety-, stage-, and environment-dependent responses to rhizobial inoculation. They do not demonstrate that all inocula are beneficial, as illustrated by the second-place composite rank of CK for ADN, nor do they establish direct nitrogen fixation or saline-soil remediation. Future work should combine replicated multi-site trials with pre-sowing soil characterization, strain-tracking, direct nitrogen-fixation assays, and factorial or mixed-effects analyses.

### 4.3 Limitations

This study has six principal limitations. First, the field trial used three replicate blocks and was conducted in one growing season, limiting power and generalizability. Second, the one-way analyses compare strains within variety and stage but do not test factorial interactions or repeated measures. Third, a pre-sowing field-soil baseline and complete date-specific management records were unavailable. Fourth, nitrogen fixation was inferred from indirect indicators and was not measured directly. Fifth, 16S rRNA gene similarity supports only provisional affiliation of the isolates. Sixth, the TOPSIS weight vector and normalized decision matrix were not available in the archived analysis records, so the reported rankings require verification against the original analysis files.

## 5 Conclusion

A greenhouse screen of 14 field isolates and one commercial inoculant identified six contrasting strains for field evaluation with three alfalfa varieties. The reported TOPSIS analysis ranked CY3+XBC, AH+SJN, and ADN+SWL first within their respective varieties, whereas individual traits often favored different strains and growth stages. In particular, CY3+XH produced the largest reported flowering-stage biomass response, and CK ranked second for ADN in the composite analysis. The study therefore identifies candidate variety–strain combinations with context-dependent agronomic performance rather than universally beneficial inocula. Because direct nitrogen fixation, strain persistence, field microbial communities, and a pre-sowing field-soil baseline were not measured, the results do not establish mechanisms of action or demonstrate remediation of saline soil. Additional multi-site validation with stronger replication and direct functional measurements is required before agronomic recommendation.

## Data Availability

The datasets presented in this study can be found in online repositories. The names of the repository/repositories and accession number(s) can be found in the article/supplementary material.
